# Towards efficient IoT communication for smart agriculture: A deep learning framework

**DOI:** 10.1371/journal.pone.0311601

**Published:** 2024-11-21

**Authors:** Ghada Alturif, Wafaa Saleh, Alaa A. El-Bary, Radwa Ahmed Osman

**Affiliations:** 1 Department of Social Work, College of Humanities and Social Sciences, Princess Nourah bint Abdulrahman University, Riyadh, Saudi Arabia; 2 School of Computing, Engineering and the Built Environment, Edinburgh Napier University, Edinburgh, Scotland, United Kingdom; 3 College of Engineering, Basic and Applied Science Institute, Arab Academy for Science, Technology and Maritime Transport, Alexandria, Egypt; 4 National Committee for Mathematics, Academy of Scientific Research and Technology, Cairo, Egypt; 5 Council of Future Studies and Risk Management, Academy of Scientific Research and Technology, Cairo, Egypt; Beijing Technology and Business University, CHINA

## Abstract

The integration of IoT (Internet of Things) devices has emerged as a technical cornerstone in the landscape of modern agriculture, revolutionising the way farming practises are viewed and managed. Smart farming, enabled by interconnected sensors and technologies, has surpassed traditional methods, giving farmers real-time, granular information into their farms. These Internet of Things devices are responsible for collecting and sending greenhouse data (temperature, humidity, and soil moisture) for the required destination, to provide a comprehensive awareness of environmental factors critical to crop growth. Therefore, ensuring that the received data are accurate is a challenge, thus this paper investigates the optimization of Agriculture IoT communication, proposing a complete strategy for improving data transmission efficiency within smart farming ecosystems. The proposed model intends to maximize energy efficiency and data throughput in the context of essential agricultural factors by using Lagrange optimization and a Deep Convolutional Neural Network (DCNN). The paper focus on the ideal communication required distance between IoT sensors that measure humidity, temperature, and water levels and central control systems. The investigation emphasizes the critical necessity of these data points in guaranteeing crop health and vitality. The proposed technique strives to improve the performance of agricultural IoT communication networks through the integration of mathematical optimization and cutting-edge deep learning. This paradigm change emphasizes the inherent link between precise achievable data rate and energy efficiency, resulting in resilient agricultural ecosystems capable of adjusting to dynamic environmental conditions for optimal crop output and health.

## Introduction

Internet of Things (IoT)-based wireless technologies have grown significantly in a number of industries in recent years. IoT is a network that enables autonomous communication between physical objects, machinery, sensors, and other things [1, 2]. Wireless devices are being used in the agriculture sector to use modern technology and improve cost management and farming productivity [3, 4]. Smart IoT devices are used in precision agriculture to monitor crop conditions at different growth stages and for remote sensing [5, 6]. Agriculture, which emphasizes the need of efficiently managing the water resources for plants, crops, and ensuring the survival of agricultural land, is one of the most important economic sectors in many countries [7, 8].

When it comes to using precision agriculture, one of the most popular technologies is sensor systems [9]. Utilising the data aggregation and transmission capabilities of sensors, remote sensing approaches have begun to communicate with Internet of Things devices for autonomous activities. Technologies include transportation, healthcare, the military, mobile phones, and home appliances are made possible by a number of real-time scenarios investigating machine learning approaches with sensors [10, 11]. Many environmental changes in the modern period impact crop and field conditions; IoT-based technologies help farmers increase productivity while reducing expenditures. The growth of smart agriculture is supported by the integration of current wireless communications technologies with cloud platforms, which may raise production productivity and improve product quality [12, 13].

However, in terms of sensing, identification, transmission, monitoring, and feedback capabilities, agriculture-related operations can be correctly carried out utilising a more dependable and sustainable method [14]. Network integrity is achieved and authentic functionalities are performed in a distributed way by secured technologies [15]. However, effective and lightweight communication paradigms require agriculture systems to have strong machine learning model functionalities. Until it is received on authorized storage and processing systems, the private agriculture data must be reliable and shielded from unwanted access [16]. It worth be mentioned the importance of fifth-generation (5G) networks ushers in a strong strategy for IoT in different situations [17]. Several 5G-enabled strategies have been given to improve the capabilities of IoT devices different circumstances [18, 19]. Furthermore, machine learning and artificial intelligence optimization algorithms play a vital role in increasing system performance by providing different solutions for models with a variety of characteristics and sectors [20, 21]. These advancements strengthen communication networks, assuring the rapid and dependable flow of critical data for timely and effective response.

IoT devices in smart agriculture often send data to the gateway or other places, sharing the 5G spectrum with D2D or cellular user equipment (CUE) connection. Interference may arise due to this shared spectrum, which could affect the dependability and efficiency of IoT connectivity. Interference in agricultural settings can result in data integrity being compromised, which can impact important decisions about crop management and access to resources. In order to improve the effectiveness and reliability of IoT communication in smart agriculture, this work suggests an novel 1D-CNN and Lagrange optimization-based approach. In order to provide a reliable and efficient transmission of agricultural data, the main goal is to reduce interference difficulties at the gateway or destination. Among this article’s major contributions are:

- A Lagrange optimization problem is built in order to determine the communication reliability of IoT devices and Gateway in smart agriculture. For increased IoT communication efficiency, this method uses a one-dimensional convolutional neural network (1D-CNN)

- The suggested approach, which is especially designed for use in smart agriculture applications, attempts to maximize communication inside IoT networks. In order to guarantee the dependable transfer of data under a variety of environmental circumstances, this optimization entails figuring out the required distance between IoT devices and Gateways. Furthermore, other variables that impact system performance are taken into account as alternative parameters. These variables include path loss, the necessary signal-to-interference-plus-noise ratio (*SINR*_*t*_*h*), transmission power, and the existence of possible interfering devices.

- IoT transmission devices can anticipate the ideal transmission distance between IoT devices and Gateways using a deep learning model that takes into consideration channel circumstances. This predictive feature ensures that patient data is received accurately and reliably in smart agriculture.

- The usefulness of the suggested technology in smart agriculture is assessed by examining the achievable data rate and energy efficiency across various environmental factors. This evaluation took into account transmission power, needed signal-to-interference-plus-noise ratio (*SINR*_*t*_*h*), and different interference transmission ranges. These findings contribute to the optimisation of IoT networks in agricultural contexts.

The structure of the paper is as follows: The details of the the proposed strategy are revealed after related work. Next, the the analytical and experimental studies for the proposed strategy are examined. Finally, a summary wraps up the paper.

## Related work

IoT communication is important since it allows for smooth connectivity across numerous devices, sensors, and agricultural equipment. This network’s allows for real-time data transmission, providing farmers with vital insights for precision farming, resource optimisation, and better decision-making. In smart farming, [22] developed a machine learning-based smart optimisation model for reliable and quality-aware sustainable agriculture. It optimised network parameters using intelligent devices, performance analysis, and blockchain-based security, which were proven through simulations and testing. Furthermore, [23] used IoT technologies to improve smart agriculture by suggesting optimised smart irrigation systems. Simulations using Network Simulator-2 (NS2) using Hierarchy Shuffled Shepherd Clustering (HSSC) and Emperor Penguin Jellyfish Optimizer (EPJO) revealed significant gains in energy efficiency and network lifetime when compared to previous approaches. Additionally, [24] proposed an Internet of Things-based smart agricultural system for India, with a focus on autonomous irrigation and insect detection. It accurately anticipated water needs and identified plant illnesses using machine learning techniques, obtaining an 84% accuracy. Moreover, [25] examined how IoT improves smart farming by monitoring soil and detecting pests with wireless sensors. For dependable information dispersion, the suggested IoT-based Wireless Sensor Network (WSN) prioritised efficient data collecting and cluster head selection.

In terms of energy efficiency for IoT networks, particularly smart agriculture network. The study presented in [26] looked at rural farms’ energy inefficiency as well as the sluggish adoption of renewable energy and resource management systems. Current renewable energy sources have been deemed insufficient for effective energy management. The report described a developed system that was applied in a farm in central Portugal, with an emphasis on integrated energy control. Solar harvesting and multi-access edge computing (MEC) were introduced in [27] for long-term monitoring in IoT-based smart agriculture. It improved network computations and energy efficiency by optimising resource scheduling and computation offloading to maximize capacity under solar energy constraints. Furthermore, [28] noted concerns about increased energy import dependency in agriculture and emphasises the importance of resource and energy allocation for improved productivity. The suggested naïve multi-phase resource allocation algorithm seeks to improve energy efficiency in dynamic agricultural situations. Additionally, [29] proposed a complete methodology for interference reduction in smart homes, emphasizing the confluence of deep learning and mathematical optimization to improve data reception reliability.

A novel and secure method for obtaining data from Internet of Things devices is proposed in [30]. SEED enabled improved throughput and energy efficiency in contrast to current methodologies by using MD5 hashing to assure data integrity and fixing network difficulties via aggregator node upgrades. Furthermore, [31] examined data transfer challenges and offered an energy-efficient Massive MIMO-NOMA IoT network for communications beyond 5G. The proposed method outperformed previous algorithms in terms of user fairness, convergence, and energy efficiency by employing sequential convex approximation and fractional programming. In addition, [32] proposed an interference control strategy to optimise 5G cellular networks and IoT. It improved crucial QoS measures such as energy economy and system reliability by decreasing interference via Lagrange optimisation, Moreover, [33] addressed interference concerns in the coexistence of 5G and IoT. It suggested a distributed deep learning model for optimising communication distances, improving throughput and energy efficiency while decreasing interference. Furthermore, [34] suggested a method for improving IoE network performance through Lagrange optimization and deep learning. It optimized transmission power for efficiency and throughput, while minimizing interference. A deep learning network predicted optimal transmission power using Lagrange optimization data, which was validated by testing.

This study addresses a crucial crop monitoring challenge in order to improve the IoT network for smart agriculture. The goal is to use the Internet of Things (IoT) to improve connectivity between farmers and agricultural systems through effective communication. In particular, the ideal range for Internet of Things communication is established when there is a chance of interference from other devices using the same frequency range. The goal is to pinpoint the necessary components and setups to enhance IoT connectivity in intelligent farming. The suggested approach combines a deep learning model with an analytical optimization technique to overcome this difficulty. The method teaches agricultural devices to dynamically modify their proximity for optimal monitoring by utilizing a distributed deep learning model within the IoT network. A comparative analysis highlighting the unique characteristics of the proposed model over previous research efforts is shown in Table 1.

**Table 1:** Comparison between different related works and the proposed model.

	Technique used	Optimization problem	Deep learning technique	Metric for system evaluation	Investigation scenario
[22]	Machine learning and blockchain-based security principles	Smart optimization model for reliable and quality-aware sustainable agriculture	N/A	Network parameters (e.g., communication interference)	Validation through simulations and experiments in smart farming systems
[23]	Hierarchy Shuffled Shepherd Clustering (HSSC) and Emperor Penguin Jellyfish Optimizer (EPJO)	Optimized intelligent smart irrigation systems for energy management	N/A	Energy consumption, network lifetime, and delay	Validation through simulation on Network Simulator-2 (NS2) and comparison with conventional methods
[24]	IoT-based smart farming system with machine learning	Automatic irrigation and plant disease detection	K-Nearest Neighbour (K-NN) and Support Vector Machine (SVM)	Classification accuracy (84%)	Monitoring, analyzing, assessing, and controlling agricultural fields for irrigation and disease detection
[25]	IoT-based WSN framework with signal-to-noise ratio (SNR) and linear congruential generator	Reliable and efficient information diffusion in smart agriculture	N/A	System throughput (16.3% improvement), packet drop ratio (36.3% reduction), network latency (12.4% reduction), energy consumption (18% reduction), and routing overheads (19% reduction)	Simulation of the proposed framework compared to other solutions
[26]	Integrated energy control with renewable energy sources	Energy management and reduction of grid energy consumption	N/A	83.2% reduction in energy from the grid, 5527 kg CO2 savings, and return on investment (ROI) of 8 years	Implementation and evaluation of a solution in a farm in central Portugal
[27]	Solar harvesting and multiaccess edge computing (MEC)	Resource scheduling and computation offloading strategy to maximize computation capacity under solar energy constraints	N/A	Efficiency in using solar energy, network energy efficiency, and sustainable agricultural WSN performance	Simulation of the proposed multiply-iterated decoupling optimization algorithm for solar-powered MEC-enabled WSNs
[28]	Naive multi-phase resource allocation algorithm	Resource allocation to enhance agricultural energy efficiency	N/A	Energy efficiency and effective utilization of agricultural resources	Addressing computational complexities in traditional data fusion algorithms for dynamic agricultural environments
[29]	Adaptive communication protocols and interference management algorithms	Minimizing interference in smart home environments by optimizing resource allocation	Deep learning for predicting and adapting to interference patterns	Data reception performance, signal quality, and robustness in various smart home situations	Evaluation of a hybrid strategy combining deep learning with optimization models for interference reduction in smart homes
[30]	Secure and energy-efficient data-collection method (SEED)	Path discovery, fault tolerance, congestion, and load balancing in IoT networks	N/A	Energy efficiency, data integrity, throughput	Evaluation of SEED method using MD5 hashing for data integrity and a unique path discovery algorithm to improve network performance
[31]	Energy-efficient Massive MIMO-NOMA IoT network	Power consumption (non-convex function), quality of service	N/A	Convergence, energy efficiency, user fairness	Implementation and evaluation of Massive MIMO-NOMA for B5G IoT networks, addressing power consumption and quality of service with iterative branch and bound techniques
[32]	Interference control model using Lagrange optimization	Interference reduction, energy efficiency, reliability	N/A	System reliability, throughput, energy efficiency	Control interference in IoT and cellular networks, optimizing performance metrics through Lagrange optimization in 5G systems
[33]	Interference avoidance distributed deep learning model	Interference reduction, throughput maximization, energy efficiency	Deep learning for interference prediction	Mean absolute error (MAE), root mean squared error (RMSE), system throughput, energy efficiency	Predicting optimal distances for IoTD-D, CUE-IoTG, BS-IoTD, and IoTG-CUE to improve throughput and energy efficiency while managing interference
[34]	Lagrange Optimization and distributed deep learning model	Power optimization, interference control, energy efficiency, system throughput	Deep learning for transmission power prediction	Energy efficiency (EE), overall system throughput (S)	Predicting optimal transmission power for uplink and downlink data communication to enhance energy efficiency and system throughput while controlling interference
Proposed model	Lagrange Optimization and Deep Convolutional Neural Network (DCNN)	Communication distance optimization, energy efficiency, data throughput	Deep Convolutional Neural Network (DCNN)	Energy efficiency, data throughput	Maximizing energy efficiency and data throughput in agricultural IoT communication by optimizing the communication distance between sensors and central control systems

## Proposed model

This section presents an analytical optimization technique that outlines a recommended approach to enhance the gateway-IoT sensor connection in smart farming. Next, a strong deep neural network architecture is shown, meant for real-world implementation in Internet of Things networks, validating the dataset generated by the analytically proposed model.

### System model and problem formulation

**Fig 1:**
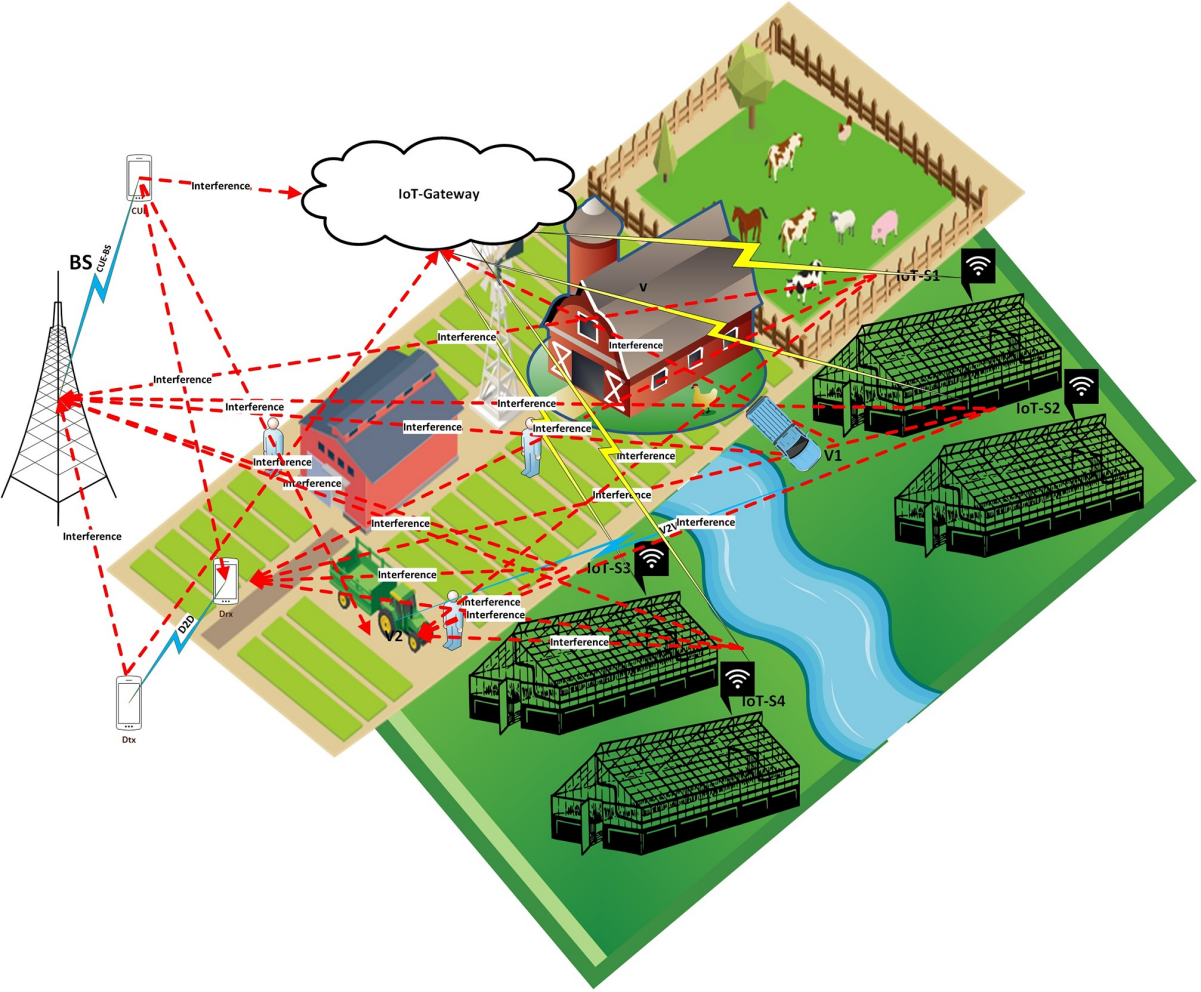
Proposed system model.

For the suggested approach, it is assumed that a smart farm consists of numerous IoT sensors are installed throughout the smart farm to monitor environmental factors such as humidity, temperature, and water levels. These sensors wirelessly transfer the collected data to a central IoT gateway. IoT gateway acts as a hub that aggregates data from all IoT sensors. It then processes this data locally and transmits it to remote servers or the cloud for further analysis and decision-making. As shown in Fig 1, the spectrum where IoT-sensors send data is shared by *C* number of CUEs, base station (BS), *D* number of D2D, which consists of transmitting devices (Dtx) and receiving devices (Drx), and *V* number of V2V, which consists of transmitting vehicles (Vtx) and receiving vehicles (Vrx). CUEs are typical mobile phones or communication devices that link to a traditional cellular service provider’s base station (BS). CUEs and IoT sensors share the same frequency range of operation. D2D communication helps lower network latency and congestion. Similar to D2D, V2V communication involves transmitting vehicles (Vtx) and receiving vehicles (Vrx) that exchange data directly. This setup is useful for applications involving autonomous or connected vehicles within the farm. This includes devices labelled as transmitting devices (Dtx) and receiving devices (Drx) that communicate directly with each other without routing through the BS. Communication scenarios include: (i) transmitting data from IoT sensors to an IoT gateway; (ii) regular cellular communication in which CUEs connect with BS; (iii) Dtx and Drx communicating D2D; and (iv) Vtx and Vrx communicating V2V. When several devices use the same spectrum and broadcast at the same time, interference in the system results. Interference may arise, for instance, if data is transmitted by a CUE, Vtx, or Dtx in the same frequency that IoT sensors use to talk to the gateway. This transmission overlap has the potential to lower data communication reliability and deteriorate signal quality. The suggested model uses a one-dimensional Convolutional Neural Network (1D-CNN) in conjunction with Lagrange optimisation to dynamically modify important communication parameters as transmission power, distances between devices, and signal-to-interference-plus-noise ratio (SINR). With the help of this adaptive technique, network performance may be optimised in real time, lowering interference and improving the dependability of IoT sensor data transfer. The suggested methodology’s main objective is to maximise the IoT system’s overall performance in smart farms. This is accomplished by optimizing the transmission power and other characteristics of D2D devices, V2V networks, CUEs, and IoT sensors in order to maximise Energy Efficiency (EE). The optimisation seeks to minimise power consumption while achieving the necessary SINR. Moreover, increasing the overall possible data throughput through the optimization of communication channels between gateways, IoT sensors, and other devices. The model accounts for a number of factors, such as interference levels, transmission power limitations, and SINR, to guarantee the best possible data throughput in a variety of environmental scenarios. The maximum energy efficiency (*EE*) and maximum achievable data rate (*R*) can be expressed as:

$$\begin{array}{c} \begin{array}{cc} \text{Maximize}\; & \sum_{i=1}^{I}\sum_{c=1}^{C}\sum_{d=1}^{D}\sum_{v=1}^{V}{EE}_{i,c,d,v}\\ \text{Subject}\;\text{to}\; & {EE}_{i,c,d,v}≔f_{1}\left(SINR_{IG},P_{C},P_{D},P_{V}\right)\\ & \left\{SINR_{IG}\geq SINR_{th},\;P_{C}\leq P_{Cmax},\;P_{D}\leq P_{Dmax},\;P_{V}\leq P_{Vmax}\right\} \end{array} \end{array}$$ (1)

$$\begin{array}{c} \begin{array}{cc} \text{Maximize}\; & \sum_{i=1}^{I}\sum_{c=1}^{C}\sum_{d=1}^{D}\sum_{v=1}^{V}R_{i,c,d,v}\\ \text{Subject}\;\text{to}\; & R_{i,c,d,v}≔f_{2}\left(SINR_{IG},P_{C},P_{D},P_{V}\right)\\ & \left\{SINR_{IG}\geq SINR_{th},\;P_{C}\leq P_{Cmax},\;P_{D}\leq P_{Dmax},\;P_{V}\leq P_{Vmax}\right\} \end{array} \end{array}$$ (2)

In the context of the optimization problem, the total achievable data rate is denoted by *R*_*i*,*c*,*d*,*v*_, while the system energy efficiency is denoted by *EE*_*i*,*c*,*d*,*v*_. These measures are related to the *v* − *th* path between V2V, the *d* − *th* path between D2D devices, the *k* − *th* path between CUE and BS, and the *i* − *th* path between IoT-sensors and gateways. The symbols *SINR*_*th*_ and *SINR*_*IG*_ represent the required system signal-to-interference-plus-noise ratio and signal-to-interference-plus-noise ratio for the Internet of Things to gateway connection, respectively. Similarly, *P*_*C*_ and *P*_*Cmax*_ indicate the CUE’s transmission power and maximum transmission power, whereas *P*_*D*_ and *P*_*Dmax*_ reflect the D2D communication link’s transmission power and maximum transmission power. Finally, *P*_*V*_ and *P*_*Vmax*_ signify the maximum transmission power and transmission power of the V2V communication link, respectively.

Non-orthogonal multiple access (NOMA) is chosen as the appropriate access method in the proposed paradigm [35, 36] to facilitate the broad implementation of IoT-sensors, CUE, D2D, and V2V for smart farms and to allow them concurrent access to the channel. Furthermore, the proposed model assumes a Rayleigh fading channel with additive white Gaussian noise (AWGN) [37]. Furthermore, the model assumes statistical independence between the channel fading coefficients for different transmission connections. As a result, the network’s energy efficiency (*EE*) and possible data rate (*R*) can be stated as follows:

$$\begin{array}{c} \begin{array}{c} EE=\frac{R_{IG}}{P_{I}+P_{o}}+\frac{R_{CB}}{P_{C}+P_{o}}+\frac{R_{DD_{rx}}}{P_{D}+P_{o}}+\frac{R_{VV_{rx}}}{P_{V}+P_{o}} \end{array} \end{array}$$ (3)

$$\begin{array}{c} \begin{array}{c} R=R_{IG}+R_{CB}+R_{DD_{rx}}+R_{VV_{rx}} \end{array} \end{array}$$ (4)

where *R*_*IG*_, *R*_*CB*_, $R_{DD_{rx}}$, and $R_{VV_{rx}}$, respectively, represent the achievable data rates for IoT-sensors and gateways, CUE-BS link, D2D communication connection, and V2V communication link. The variables *P*_*I*_ and *P*_*o*_ denote the IoT transmission power and internal circuit power consumption. As a result, the expressions *R*_*IG*_, *R*_*CB*_, $R_{DD_{rx}}$, and $R_{VV_{rx}}$ are:

$$\begin{array}{c} \begin{array}{c} R_{IG}=B\;{\text{log}}_{2}(1+\frac{P_{I}H_{IG}}{\sum_{k=1}^{K}\;P_{C}H_{C_{k}G}+\sum_{d=1}^{D}\;P_{D}H_{D_{d}G}+\sum_{v=1}^{V}\;P_{V}H_{V_{v}G}+N}) \end{array} \end{array}$$ (5)

$$\begin{array}{c} \begin{array}{c} R_{CB}=B\;{\text{log}}_{2}(1+\frac{P_{C}H_{CB}}{\sum_{i=1}^{I}\;P_{I}H_{I_{i}B}+\sum_{d=1}^{D}\;P_{D}H_{D_{d}B}+\sum_{v=1}^{V}\;P_{V}H_{V_{v}B}+N}) \end{array} \end{array}$$ (6)

$$\begin{array}{c} \begin{array}{c} R_{DD_{rx}}=B\;{\text{log}}_{2}(1+\frac{P_{D}H_{DD_{rx}}}{\sum_{i=1}^{I}\;P_{I}H_{I_{i}D_{rx}}+\sum_{k=1}^{K}\;P_{C}H_{C_{k}D_{rx}}+\sum_{v=1}^{V}\;P_{V}H_{V_{v}D_{rx}}+N}) \end{array} \end{array}$$ (7)

$$\begin{array}{c} \begin{array}{c} R_{VV_{rx}}=B\;{\text{log}}_{2}(1+\frac{P_{V}H_{VV_{rx}}}{\sum_{i=1}^{I}\;P_{I}H_{I_{i}V_{rx}}+\sum_{k=1}^{K}\;P_{C}H_{C_{k}V_{rx}}+\sum_{d=1}^{D}\;P_{D}H_{D_{d}V_{rx}}+N}) \end{array} \end{array}$$ (8)

where the channel gain coefficient between IoT-sensors and gateways (G), Cellular equipment deices (CUE) and gateways, transmitted device (Dtx) and gateways, and transmitted vehicle (Vtx) and gateways are *H*_*IG*_, *H*_*C*_*k*_*G*_, *H*_*D*_*d*_*G*_ and *H*_*V*_*v*_*G*_, respectively. The channel gain coefficient between Cellular equipment deices (CUE) and base station (BS), IoT-sensors and base station (BS), transmitted device (Dtx) and base station (BS), and transmitted vehicle (Vtx) and base station (BS) are represented by *H*_*CB*_, *H*_*I*_*i*_*B*_, *H*_*D*_*d*_*B*_ and *H*_*V*_*v*_*B*_, respectively. The channel gain coefficient between transmitted device (Dtx) and receiving device (Drx), IoT-sensors and receiving device (Drx), Cellular equipment deices (CUE) and receiving device (Drx), and transmitted vehicle (Vtx) receiving device (Drx) are represented by $H_{DD_{rx}}$, $H_{I_{i}D_{rx}}$, $H_{C_{k}D_{rx}}$ and *H*_*V*_*v*_*D*_, respectively. The channel gain coefficient between transmitted vehicle (Vtx) and receiving vehicle (Vrx), IoT-sensors and receiving vehicle (Vrx), CUE and receiving vehicle (Vrx), and Dtx and receiving vehicle (Vrx) are represented by *H*_*VV*_, $H_{I_{i}V_{rx}}$, $H_{C_{k}V_{rx}}$ and $H_{D_{d}V_{rx}}$, respectively. In this case, *N* and *B* represent the noise power and channel system bandwidth, respectively.

The main goal of the proposed methodology is to maximize the total achievable data rate (*R*) and overall energy efficiency (*EE*) of the Internet of Things network in smart agriculture under different environmental scenarios, as demonstrated by Eqs 1 and 2. Under the restriction that the signal-to-interference-plus-noise ratio (SINR) between Internet of Things devices and gateways (*SINR*_*IG*_) must either meet or above a predetermined threshold (*SINR*_*th*_), Energy Efficiency (EE) is optimized. Furthermore, the maximum limits (*P*_*Dmax*_, *P*_*Dmax*_, *P*_*Vmax*_) for the transmission powers of the Cellular User Equipment (CUE), Device-to-Device (D2D) links, and Vehicle-to-Vehicle (V2V) links must be adhered to. Furthermore, Similar restrictions apply to the maximum achievable data rate optimization as they do to energy efficiency, guaranteeing dependable communication and peak network performance. The Lagrange multipliers method was applied to the optimization problems for EE and R in order to accommodate these limitations. The following Eqs 1 and 2 is the formulation of the Lagrangian functions for the optimization problems:

$$\begin{array}{c} \begin{array}{c} L\left({SINR}_{IG},P_{C},P_{D},P_{V},\lambda_{1},\lambda_{2},\lambda_{3},\lambda_{4}\right)=EE+\lambda_{1}\left({SINR}_{IG}-{SINR}_{th}\right)\\ +\lambda_{2}\left(P_{C}-P_{Cmax}\right)++\lambda_{3}\left(P_{D}-P_{Dmax}\right)+\lambda_{4}\left(P_{V}-P_{Vmax}\right). \end{array} \end{array}$$ (9)

$$\begin{array}{c} \begin{array}{c} L\left({SINR}_{IG},P_{C},P_{D},P_{V},\mu_{1},\mu_{2},\mu_{3},\mu_{4}\right)=R+\mu_{1}\left({SINR}_{IG}-{SINR}_{th}\right)\\ +\mu_{2}\left(P_{C}-P_{Cmax}\right)+\mu_{3}\left(P_{D}-P_{Dmax}\right)+\mu_{4}\left(P_{V}-P_{Vmax}\right). \end{array} \end{array}$$ (10)

The non-negative Lagrangian multipliers are designated by the symbols λ_1_, λ_2_, λ_3_, λ_4_, *μ*_1_, *μ*_2_, *μ*_3_, and *μ*_4_. The values of λ_1_, λ_2_, λ_3_, and λ_4_ must be determined by calculating the derivative of Eq 9 with reference to *P*_*I*_, *P*_*C*_, *P*_*D*_, and *P*_*V*_ to fulfil the conditions of the optimization problem for energy efficiency (*EE*). These multipliers help to adjust the optimization problem by penalizing any violation of the constraints. An increased Lagrange multiplier value signifies a more robust impact of the associated constraint on the optimization procedure. As a result, λ_1_, λ_2_, λ_3_, and λ_4_ can be calculated as follows:

$$\begin{array}{c} \begin{array}{ccc} \lambda_{1} & =\frac{B\cdot X_{1}}{\left(P_{I}+P_{o}\right)}+\frac{B\cdot {\text{log}}_{2}\left(1+P_{I}\cdot X_{2}\right)}{{\left(P_{I}+P_{o}\right)}^{2}\cdot X_{2}} & \;+\frac{B\cdot X_{3}\cdot X_{4}}{\left(P_{C}+P_{o}\right)\cdot X_{2}}+\frac{B\cdot X_{5}\cdot X_{6}}{\left(P_{D}+P_{o}\right)\cdot X_{2}}\\ & \;+\frac{B\cdot X_{7}\cdot X_{8}}{\left(P_{V}+P_{o}\right)\cdot X_{2}} \end{array} \end{array}$$ (11)

$$\begin{array}{c} \begin{array}{cc} \lambda_{2} & =\frac{B\cdot X_{1}}{\left(P_{I}+P_{o}\right)}\cdot \frac{P_{I}\sum_{k=1}^{K}\;H_{c_{k}G}\cdot X_{2}}{C}+\frac{B\cdot {\text{log}}_{2}\left(1+P_{C}\cdot X_{9}\right)}{{\left(P_{C}+P_{o}\right)}^{2}}-\frac{B\cdot X_{3}\cdot X_{9}}{\left(P_{C}+P_{o}\right)}\\ & \;+\frac{B\cdot X_{5}\cdot X_{10}}{\left(P_{D}+P_{o}\right)}+\frac{B\cdot X_{7}\cdot X_{11}}{\left(P_{V}+P_{o}\right)}+\lambda_{1}\left(\frac{P_{I}\sum_{k=1}^{K}\;H_{C_{k}G}\cdot X_{2}}{C}\right) \end{array} \end{array}$$ (12)

$$\begin{array}{c} \begin{array}{cc} \lambda_{3} & =\frac{B\cdot X_{1}}{\left(P_{I}+P_{o}\right)}\cdot \frac{P_{I}\sum_{l=1}^{L}\;H_{D_{d}G}\cdot X_{2}}{C}+\frac{B\cdot X_{3}\cdot X_{12}}{\left(P_{C}+P_{o}\right)}+\frac{B\cdot {\text{log}}_{2}\left(1+P_{D}X_{13}\right)}{{\left(P_{D}+P_{o}\right)}^{2}}\\ & \;-\frac{B\cdot X_{5}\cdot X_{13}}{\left(P_{D}+P_{o}\right)}+\frac{B\cdot X_{7}\cdot X_{14}}{\left(P_{V}+P_{o}\right)}+\lambda_{1}\left(\frac{P_{I}\sum_{d=1}^{D}\;H_{D_{d}G}\cdot X_{2}}{C}\right) \end{array} \end{array}$$ (13)

$$\begin{array}{c} \begin{array}{cc} \lambda_{4} & =\frac{B\cdot X_{1}}{\left(P_{I}+P_{o}\right)}\cdot \frac{P_{I}\sum_{v=1}^{V}\;H_{V_{v}G}\cdot X_{2}}{C}+\frac{B\cdot X_{3}\cdot X_{15}}{\left(P_{C}+P_{o}\right)}+\frac{B\cdot X_{5}\cdot X_{16}}{\left(P_{D}+P_{o}\right)}+\\ & \;\frac{B\cdot {\text{log}}_{2}\left(1+P_{V}X_{17}\right)}{{\left(P_{V}+P_{o}\right)}^{2}}-\frac{B\cdot X_{7}\cdot X_{17}}{\left(P_{V}+P_{o}\right)}+\lambda_{1}(\frac{P_{I}\sum_{v=1}^{V}\;H_{V_{v}G}\cdot X_{2}}{C}) \end{array} \end{array}$$ (14)

where

$$\begin{array}{cc} C & =\sum_{k=1}^{K}\;P_{C}H_{C_{k}G}+\sum_{d=1}^{D}\;P_{D}H_{D_{d}G}+\sum_{v=1}^{V}\;P_{V}H_{V_{v}G}+N, \end{array}$$

$$\begin{array}{cc} X_{1} & =\frac{\sum_{k=1}^{K}\;P_{C}H_{C_{k}G}+\sum_{d=1}^{D}\;P_{D}H_{D_{d}G}+\sum_{v=1}^{V}\;P_{V}H_{V_{v}G}+N}{\sum_{k=1}^{K}\;P_{C}H_{C_{k}G}+\sum_{d=1}^{D}\;P_{D}H_{D_{d}G}+\sum_{v=1}^{V}\;P_{V}H_{V_{v}G}+N+P_{I}H_{ID}}, \end{array}$$

$$\begin{array}{cc} X_{2} & =\frac{H_{IG}}{\sum_{k=1}^{K}\;P_{C}H_{C_{k}G}+\sum_{d=1}^{D}\;P_{D}H_{D_{d}G}+\sum_{v=1}^{V}\;P_{V}H_{V_{v}G}+N}, \end{array}$$

$$\begin{array}{cc} X_{3} & =\frac{\sum_{i=1}^{I}\;P_{I}H_{I_{i}B}+\sum_{d=1}^{D}\;P_{D}H_{D_{d}B}+\sum_{v=1}^{V}\;P_{V}H_{V_{v}B}+N}{\sum_{i=1}^{I}\;P_{I}H_{I_{i}B}+\sum_{d=1}^{D}\;P_{D}H_{D_{d}B}+\sum_{v=1}^{V}\;P_{V}H_{V_{v}B}+N+P_{C}H_{CB}}, \end{array}$$

$$\begin{array}{cc} X_{4} & =\frac{P_{C}H_{CB}\sum_{i=1}^{I}\;H_{I_{i}B}}{{\left(\sum_{i=1}^{I}\;P_{I}H_{I_{i}B}+\sum_{d=1}^{D}\;P_{D}H_{D_{d}B}+\sum_{v=1}^{V}\;P_{V}H_{V_{v}B}+N\right)}^{2}}, \end{array}$$

$$\begin{array}{cc} X_{5} & =\frac{\sum_{i=1}^{I}\;P_{I}H_{I_{i}D_{rx}}+\sum_{k=1}^{K}\;P_{C}H_{C_{k}D_{rx}}+\sum_{v=1}^{V}\;P_{V}H_{V_{v}D_{rx}}+N}{\sum_{i=1}^{I}\;P_{I}H_{I_{i}D_{rx}}+\sum_{k=1}^{K}\;P_{C}H_{C_{k}D_{rx}}+\sum_{v=1}^{V}\;P_{V}H_{V_{v}D_{rx}}+N+P_{D}H_{DD_{rx}}}, \end{array}$$

$$\begin{array}{cc} X_{6} & =\frac{P_{D}H_{DD_{rx}}\sum_{i=1}^{I}\;H_{I_{i}D_{rx}}}{{\left(\sum_{i=1}^{I}\;P_{I}H_{I_{i}D_{rx}}+\sum_{k=1}^{K}\;P_{C}H_{C_{k}D_{rx}}+\sum_{v=1}^{V}\;P_{V}H_{V_{v}D_{rx}}+N\right)}^{2}}, \end{array}$$

$$\begin{array}{cc} X_{7} & =\frac{\sum_{i=1}^{I}\;P_{I}H_{I_{i}V_{rx}}+\sum_{k=1}^{K}\;P_{C}H_{C_{k}V_{rx}}+\sum_{d=1}^{D}\;P_{D}H_{D_{d}V_{rx}}+N}{\sum_{i=1}^{I}\;P_{I}H_{I_{i}V_{rx}}+\sum_{k=1}^{K}\;P_{C}H_{C_{k}V_{rx}}+\sum_{d=1}^{D}\;P_{D}H_{D_{d}V}+N+P_{D}H_{DD_{rx}}}, \end{array}$$

$$\begin{array}{cc} X_{8} & =\frac{P_{V}H_{VV_{rx}}\sum_{i=1}^{I}\;H_{I_{i}V_{rx}}}{{\left(\sum_{i=1}^{I}\;P_{I}H_{I_{i}V_{rx}}+\sum_{k=1}^{K}\;P_{C}H_{C_{k}V_{rx}}+\sum_{d=1}^{D}\;P_{D}H_{D_{d}V_{rx}}+N\right)}^{2}}, \end{array}$$

$$\begin{array}{cc} X_{9} & =\frac{H_{CB}}{\sum_{i=1}^{I}\;P_{I}H_{I_{i}B}+\sum_{d=1}^{D}\;P_{D}H_{D_{d}B}+\sum_{v=1}^{V}\;P_{V}H_{V_{v}B}+N}, \end{array}$$

$$\begin{array}{cc} X_{10} & =\frac{P_{D}H_{DD_{rx}}\sum_{k=1}^{K}\;H_{C_{k}D_{rx}}}{{\left(\sum_{i=1}^{I}\;P_{I}H_{I_{i}D_{rx}}+\sum_{k=1}^{K}\;P_{C}H_{C_{k}D_{rx}}+\sum_{v=1}^{V}\;P_{V}H_{V_{v}D_{rx}}+N\right)}^{2}}, \end{array}$$

$$\begin{array}{cc} X_{11} & =\frac{P_{V}H_{VV_{rx}}\sum_{k=1}^{K}\;H_{C_{k}V_{rx}}}{{\left(\sum_{i=1}^{I}\;P_{I}H_{I_{i}V_{rx}}+\sum_{k=1}^{K}\;P_{C}H_{C_{k}V_{rx}}+\sum_{d=1}^{D}\;P_{D}H_{D_{d}V_{rx}}+N\right)}^{2}}, \end{array}$$

$$\begin{array}{cc} X_{12} & =\frac{P_{B}H_{CB}\sum_{l=1}^{L}\;P_{D}H_{D_{l}B}}{{\left(\sum_{i=1}^{I}\;P_{I}H_{I_{i}B}+\sum_{d=1}^{D}\;P_{D}H_{D_{d}B}+\sum_{v=1}^{V}\;P_{V}H_{V_{v}B}+N\right)}^{2}}, \end{array}$$

$$\begin{array}{cc} X_{13} & =\frac{H_{DD_{rx}}}{\sum_{i=1}^{I}\;P_{I}H_{I_{i}D_{rx}}+\sum_{k=1}^{K}\;P_{C}H_{C_{k}D_{rx}}+\sum_{v=1}^{V}\;P_{V}H_{V_{v}D_{rx}}+N}, \end{array}$$

$$\begin{array}{cc} X_{14} & =\frac{P_{V}H_{VV_{rx}}\sum_{d=1}^{D}\;H_{D_{d}V_{rx}}}{{\left(\sum_{i=1}^{I}\;P_{I}H_{I_{i}V_{rx}}+\sum_{k=1}^{K}\;P_{C}H_{C_{k}V_{rx}}+\sum_{d=1}^{D}\;P_{D}H_{D_{d}V_{rx}}+N\right)}^{2}}, \end{array}$$

$$\begin{array}{cc} X_{15} & =\frac{P_{B}H_{CB}\sum_{v=1}^{V}\;P_{V}H_{V_{v}B}}{{\left(\sum_{i=1}^{I}\;P_{I}H_{I_{i}B}+\sum_{d=1}^{D}\;P_{D}H_{D_{d}B}+\sum_{v=1}^{V}\;P_{V}H_{V_{v}B}+N\right)}^{2}}, \end{array}$$

$$\begin{array}{cc} X_{16} & =\frac{P_{D}H_{DD_{rx}}\sum_{v=1}^{V}\;P_{V}H_{V_{v}D_{rx}}}{{\left(\sum_{i=1}^{I}\;P_{I}H_{I_{i}D_{rx}}+\sum_{k=1}^{K}\;P_{C}H_{C_{k}D_{rx}}+\sum_{v=1}^{V}\;P_{V}H_{V_{v}D_{rx}}+N\right)}^{2}}and \end{array}$$

$$\begin{array}{cc} X_{17} & =\frac{H_{VV_{rx}}}{\sum_{i=1}^{I}\;P_{I}H_{I_{i}V_{rx}}+\sum_{k=1}^{K}\;P_{C}H_{C_{k}V_{rx}}+\sum_{d=1}^{D}\;P_{D}H_{D_{d}V_{rx}}+N} \end{array}$$

The derivation of Eq 9 with respect to λ_1_, λ_2_, λ_3_, and λ_4_ yields the optimal required distance (*d*_*IG*_) between IoT-sensors and gateways, the optimal required CUE interfere transmission power (*P*_*C*_), the optimal required Dtx interfere transmission power (*P*_*D*_), and the optimal required Vtx interfere transmission power (*P*_*V*_). These can be found as:

$$\begin{array}{c} \begin{array}{c} d_{IG}={\left[\frac{{SINR}_{th}(\sum_{k=1}^{K}\;P_{C}H_{C_{k}G}+\sum_{d=1}^{D}\;P_{D}H_{D_{d}G}+\sum_{v=1}^{V}\;P_{V}H_{V_{v}G}+N)}{P_{I}/pl_{o}}\right]}^{-1/\alpha } \end{array} \end{array}$$ (15)

where the path loss exponent and constant path loss are expressed by *α* and *pl*_*o*_, respectively.

$$\begin{array}{c} \begin{array}{c} P_{C}=P_{C_{max}} \end{array} \end{array}$$ (16)

$$\begin{array}{c} \begin{array}{c} P_{D}=P_{D_{max}} \end{array} \end{array}$$ (17)

$$\begin{array}{c} \begin{array}{c} P_{V}=P_{V_{max}} \end{array} \end{array}$$ (18)

The values of *μ*_1_, *μ*_2_, *μ*_3_, and *μ*_4_ can be found using the derivative of Eq 10 with regard to *P*_*I*_, *P*_*C*_, *P*_*D*_, and *P*_*V*_ in order to satisfy the constraint of the optimization problem for (*R*). Then, *μ*_1_, *μ*_2_, *μ*_3_, and *μ*_4_ may represent as follows:

$$\begin{array}{c} \begin{array}{c} \mu_{1}=\frac{B\cdot X_{1}\cdot X_{2}+B\cdot X_{3}\cdot X_{4}+B\cdot X_{5}\cdot X_{6}+B\cdot X_{7}\cdot X_{8}}{X_{2}} \end{array} \end{array}$$ (19)

$$\begin{array}{c} \begin{array}{cc} \mu_{2} & =B\cdot X_{1}\left(\frac{P_{I}\sum_{k=1}^{K}\;H_{C_{k}G}\cdot X_{2}}{C}\right)-B\cdot X_{3}\cdot X_{9}+B\cdot X_{5}\cdot X_{10}+B\cdot X_{7}\cdot X_{11}\\ & \;+\lambda_{1}\left(\frac{P_{I}\sum_{k=1}^{K}\;H_{C_{k}G}\cdot X_{2}}{C}\right) \end{array} \end{array}$$ (20)

$$\begin{array}{c} \begin{array}{cc} \mu_{3} & =BX_{1}(\frac{P_{I}\sum_{l=1}^{L}\;H_{D_{d}G}\cdot X_{2}}{C})+B\cdot X_{3}\cdot X_{12}-B\cdot X_{5}\cdot X_{13}+B\cdot X_{7}\cdot X_{14}\\ & \;+\lambda_{1}(\frac{P_{I}\sum_{d=1}^{D}\;H_{D_{d}G}\cdot X_{2}}{C}) \end{array} \end{array}$$ (21)

$$\begin{array}{c} \begin{array}{cc} \mu_{4} & =BX_{1}(\frac{P_{I}\sum_{v=1}^{V}\;H_{V_{v}G}\cdot X_{2}}{C})+B\cdot X_{3}\cdot X_{15}+B\cdot X_{5}\cdot X_{16}-B\cdot X_{7}\cdot X_{17}\\ & \;+\lambda_{1}(\frac{P_{I}\sum_{v=1}^{V}\;H_{V_{v}G}\cdot X_{2}}{C}) \end{array} \end{array}$$ (22)

The optimal required interference distance (*d*_*IG*_) between IoT-sensors and the gateway, the optimal required CUE interfere transmission power (*P*_*C*_), the optimal required Dtx interfere transmission power (PD), and the optimal required Vtx interfere transmission power (*P*_*V*_) can all be obtained by deriving Eq 10 with respect to *μ*_1_, *μ*_2_, *μ*_3_, and *μ*_4_. This will make it possible to optimize the overall attainable data rate (*R*), which may be calculated with the following formula:

$$\begin{array}{c} \begin{array}{c} d_{IG}={\left[\frac{{SINR}_{th}(\sum_{k=1}^{K}\;P_{C}H_{C_{k}G}+\sum_{d=1}^{D}\;P_{D}H_{D_{d}G}+\sum_{v=1}^{V}\;P_{V}H_{V_{v}G}+N)}{P_{I}/pl_{o}}\right]}^{-1/\alpha } \end{array} \end{array}$$ (23)

$$\begin{array}{c} \begin{array}{c} P_{C}=P_{C_{max}} \end{array} \end{array}$$ (24)

$$\begin{array}{c} \begin{array}{c} P_{D}=P_{D_{max}} \end{array} \end{array}$$ (25)

$$\begin{array}{c} \begin{array}{c} P_{V}=P_{V_{max}} \end{array} \end{array}$$ (26)

### Dataset generation

The necessary datasets have been made available through MATLAB simulations, and the equations for the proposed model—which are described in Section —have been put into practice. The simulation’s parameter values are displayed in Table 2. To improve communication between IoT-sensors and gateway, the datasets will be utilized to train models that will be put on all transmitting devices.

**Table 2:** Simulation parameters.

Parameter	Value
*N*	-174 dBm/Hz [38]
*B*	10 Mbit/s [39]
*α*	4
*P*_*I*_	23 dBm [40]
*P*_*C*_	23 dBm [40]
*P*_*D*_	23 dBm [40]
*P*_*V*_	23 dBm [40]
*SINR*_*th*_	20 dB [40]
Pathloss between CUE and BS	$148+40\;{\text{log}}_{2}(\frac{d_{CB}}{\text{km}})$
Pathloss between D2D link	$128.1+37.6\;{\text{log}}_{2}(\frac{d_{DD}}{\text{km}})$
Pathloss between V2V link	$128.1+37.6\;{\text{log}}_{2}(\frac{d_{VV}}{\text{km}})$

**Fig 2:**
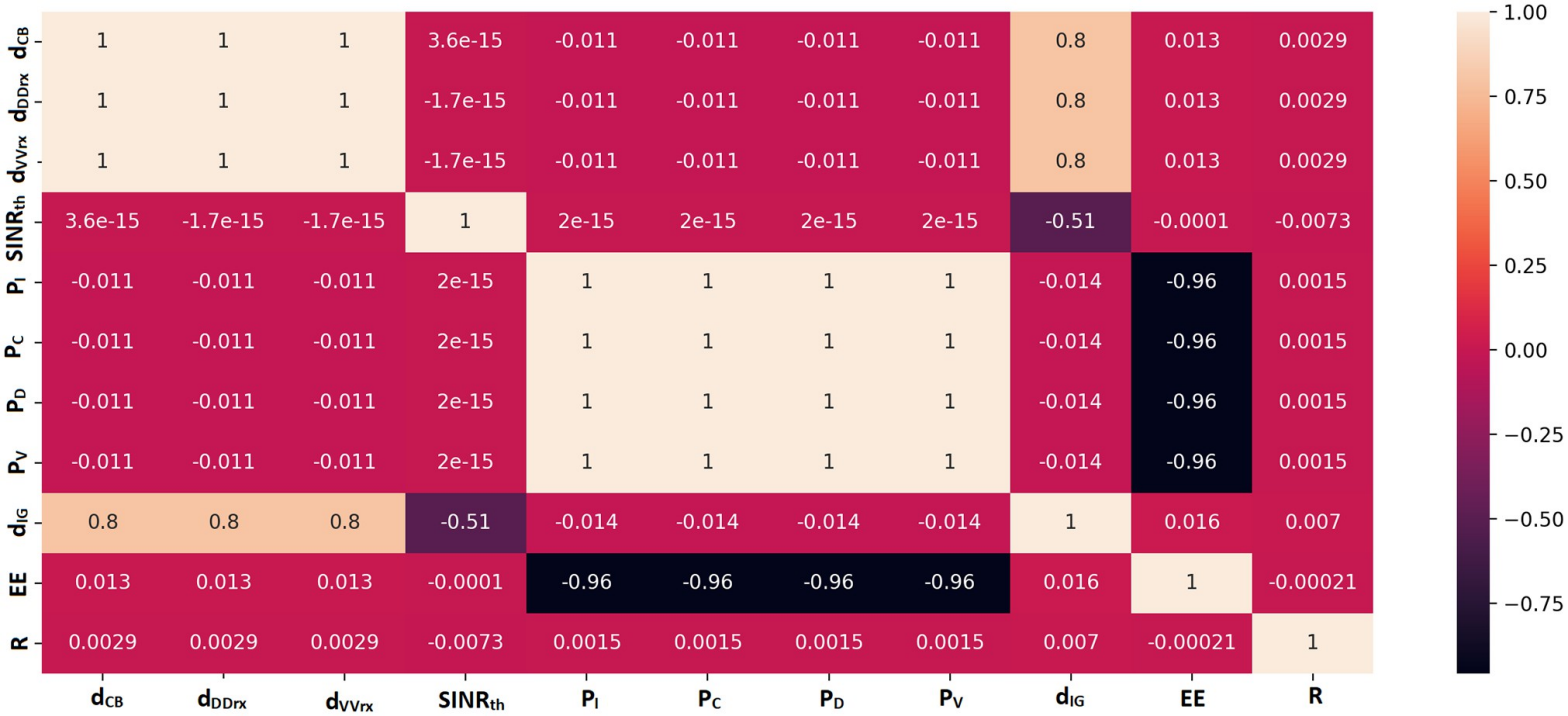
Pearson correlation coefficients of each input parameter (*d*_*CB*_, $d_{DD_{rx}}$, $d_{VV_{rx}}$, *SINR*_*th*_, *P*_*I*_, *P*_*C*_, *P*_*D*_ and *P*_*V*_) and the output (*d*_*IG*_, EE and R).

There are 44679 records in all. Each record contains a unique combination of these variables to represent the following: the distances between CUB and BS (*d*_*CB*_), Dtx and Drx (*d*_*DD*_*r*_*x*_), and Vtx and Vrx (*d*_*VV*_*r*_*x*_); the necessary signal-to-interference-plus-noise-ratio threshold (*SINR*_*th*_); the IoT device transmission power (*P*_*I*_), the CUE transmission power (*P*_*C*_), the D2D transmission power (*P*_*D*_), and the V2V transmission power (*P*_*V*_) Fig 2 shows the Pearson coefficients that illustrate the relationship between each input and output parameter. The graph shows that EE has a substantial negative correlation with the *P*_*I*_, *P*_*C*_, *P*_*D*_, and *P*_*V*_ parameters, whereas the output *d*_*IG*_ has a strong association with the *d*_*CB*_, $d_{DD_{rx}}$, and $d_{VV_{rx}}$ parameters. Furthermore, there isn’t much of a link between parameters *R* and the input parameters. Each of these variables must be used to train the deep learning model, and the results section will provide an explanation of the association’s significance.

### Proposed deep learning model

**Fig 3:**
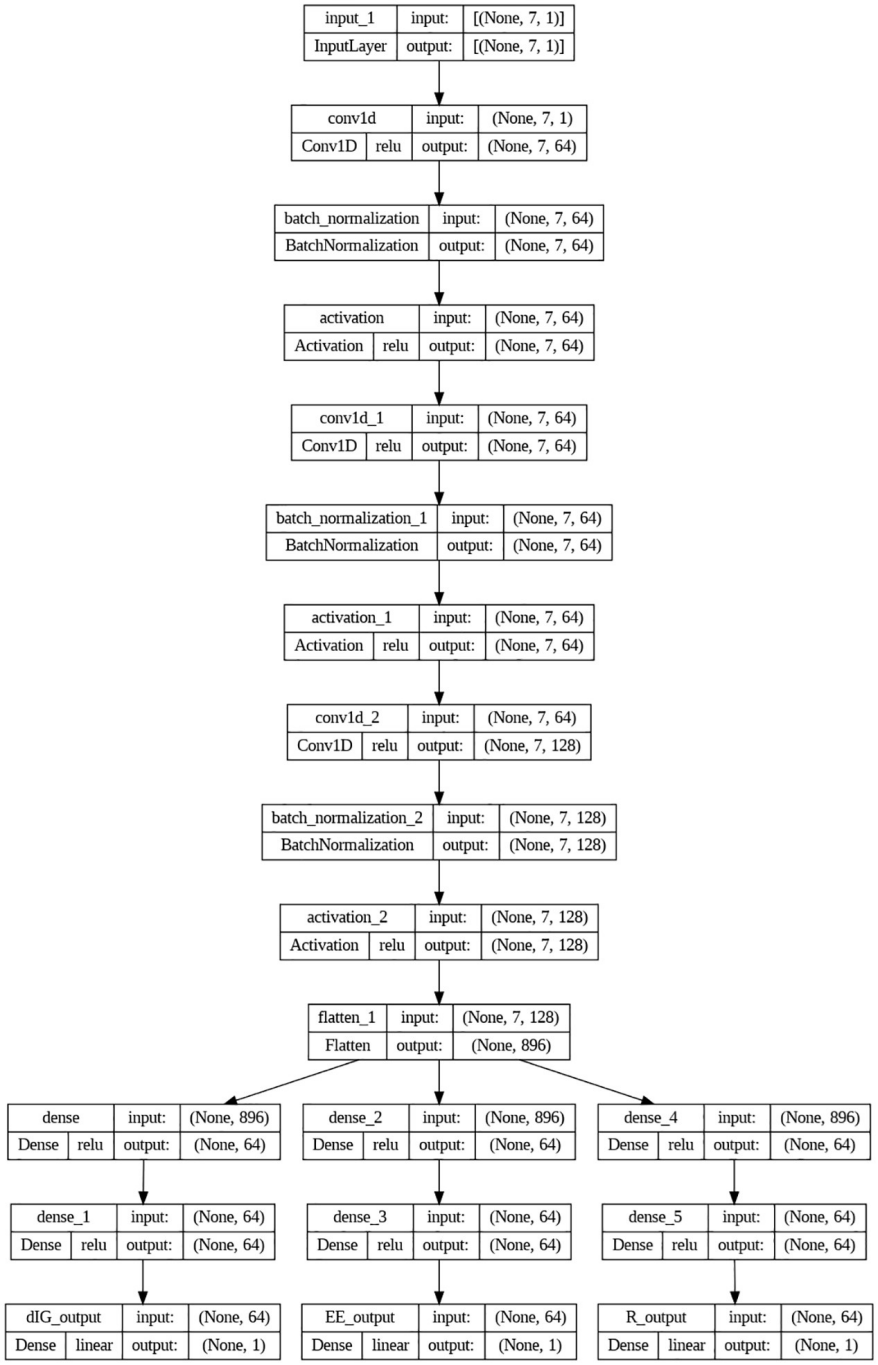
Proposed deep learning model.

In this section, the suggested deep learning model is demonstrated and explained. Before adding the variables to the recommended deep learning model, a normalization phase must be completed in order to help with the learning of the model weights. Each variable is normalized using the min-max scaling procedure before being incorporated to the model. From the final dense layer, the eight input variables, *d*_*CB*_, $d_{DD_{rx}}$, $d_{VV_{rx}}$, *SINR*_*th*_, *P*_*I*_, *P*_*C*_, *P*_*D*_, and *P*_*V*_, are used to derive the output parameters, *d*_*IG*_, *EE*, and *R*. The model has three distinct phases, namely 1D-CNN, flattening, and thick layers, as illustrated in Fig 3. The normalized input parameters are processed by three 1D-CNN layers: one with a size 1 kernel and each with 64, 64, and 128 filters.

To maintain a constant width of the output matrix, each layer of the 1D-CNN produces padded results. Next, a flattening layer receives the output from the third 1D-CNN and reformats the dimension to prepare it for input into the dense layers. Regression is produced by six dense layers that come after the flattening layer. Before choosing how many nodes to utilise for the dense layers and how many filters to employ for the 1D-CNN, a grid search was utilised to test out a number of options. The activation function for each hidden layer has been the Rectified Linear Unit (ReLU). The grid search took activation function selection into consideration and tested several methods for following the hidden layers in the proposed model. For optimal results, the output of each hidden layer was input into an activation function known as a parametric rectified linear unit, or PReLU.

The root mean square error (RMSE) and mean absolute error (MAE) loss function are the objectives of the adaptive moment (Adam) optimization used in the proposed model. Adam’s learning method allows him to acquire the required abilities. Whereas RMSE is the root square of the average of the squared disparities between real and anticipated values, MAE measures the average difference between the actual and expected values. They can be referred to as these:

$$\begin{array}{c} MAE=\frac{\sum_{j=1}^{n}\;\left|y_{j}-x_{j}\right|}{n} \end{array}$$ (27)

$$\begin{array}{c} RMSE=\sqrt{\frac{\sum_{j=1}^{n}\;{\left(y_{j}-x_{j}\right)}^{2}}{n}} \end{array}$$ (28)

where *x*_*j*_ is the predicted value, *y*_*j*_ is the actual value, and *n* is the total number of data points that were recorded. The experiments that were conducted in order to develop, validate, and test the proposed model are covered in the section that follows.

## Results and discussion

**Fig 4:**
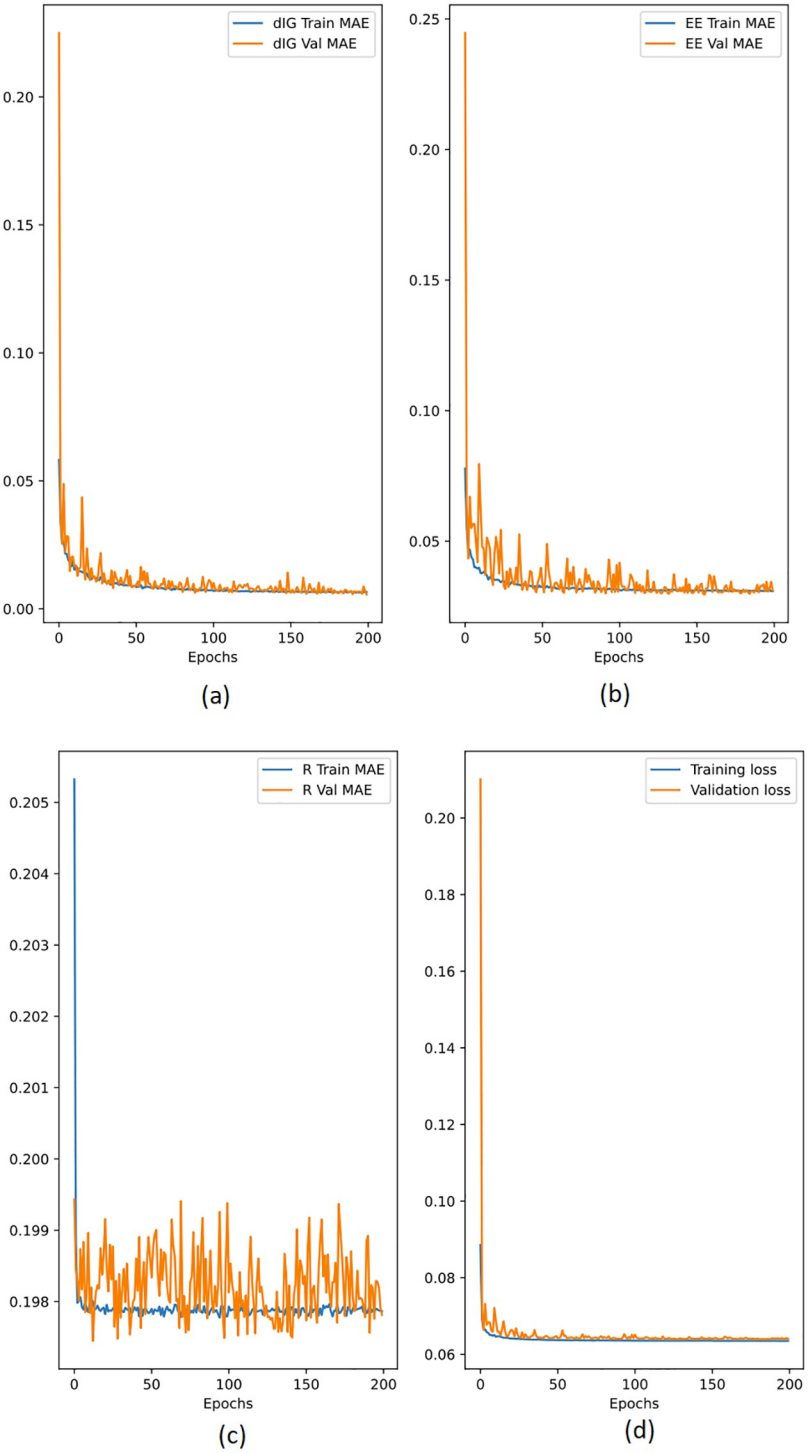
Training and validation mean absolute error generated during training the proposed model.

This section presents the performance of the suggested deep learning and analytical models. Furthermore, the effectiveness of the suggested method was assessed in terms of achievable data rate and improved energy efficiency using MATLAB and Python simulations. As seen in Fig 4, the suggested deep learning model from section is assessed and put to the test. An 80% train set and a 20% test set were created from the datasets. The needed *d*_*IG*_, *EE*, and *R* are shown as the training and validation mean absolute errors in Fig 4(a)–4(c), respectively. Since the results were not changing noticeably beyond epoch 100, all of these graphs demonstrate that additional training was not necessary. Furthermore, Fig 4(d) shows nearly similar independent training and validation errors for each output, indicating that the proposed model was neither overfitted nor underfitted. It also shows how the independent training and validation mistakes loss eventually decrease and stabilise.

**Fig 5:**
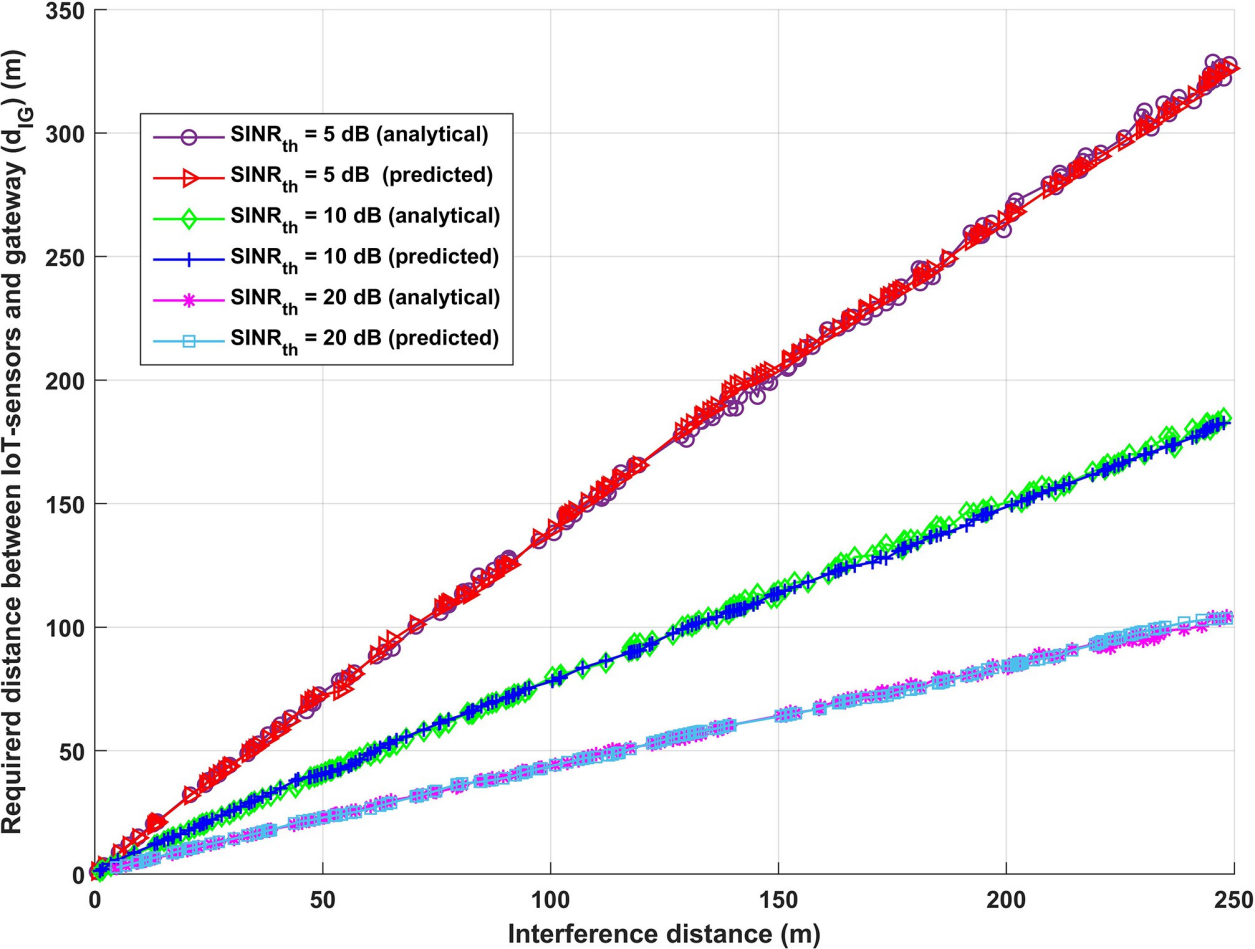
Interference distance (m) vs required distance between IoT-sensors and gateway (*d*_*IG*_).

It has been assumed for the system evaluation that the transmission power of IoT-sensors (*P*_*I*_), CUE (*P*_*C*_), D2D (*P*_*D*_), and V2V (*P*_*V*_) are equal, and that *SINR*_*t*_*h* and the transmission distance are always changing. The interference transmission lengths between any interfere transmitter and its destination (D2D, V2V, and CUE-BS connections) are shown in Fig 5 in comparison to the necessary transmission length (*d*_*I*_*G*) between IoT-sensors and gateway (G) for the deep learning and analytical models. Various values of *SINR*_*th*_, namely 5 dB, 15 dB, and 20 dB, have been investigated to evaluate the efficacy of the proposed model. Furthermore, it has been assumed that the transmission power for all IoT-sensors and interfere devices, which is 23 dBm, and the transmission distances between Dtx and Drx, as well as between Vtx and Vrx, are 1/5 of the transmission distances between CUE and BS. In the worst case scenario, high amounts of transmission power interference may affect the transfer data from IoT sensors. For each *SINR*_*th*_ supplied, and for each interfere transmission distance, as can be seen in Fig 5, there exists an ideal necessary transmission distance between IoT-sensors and G (*d*_*IG*_) in order to fulfil the required IoT system performance for both the analytical and deep learning models. For example, when *SINR*_*th*_ is 5 and the interference transmission distance is 100.7 m, the optimal necessary transmission distance between IoT-sensors and G (*d*_*IG*_) for the analytical and deep learning models, respectively, to meet IoT system performance is 103.399 m and 104.7424 m. On the other hand, with *SINR*_*th*_ = 20, the interference transmission distance is 101 m for the analytical and deep learning models, and 44.3611 m for the optimal necessary transmission distance between IoT-sensors and G (*d*_*IG*_). After comparing the two scenarios, it can be concluded that increasing *SINR*_*th*_ guarantees that the data will be sent across an efficient communication channel and that the received data is reliable enough to support decision-making while also reducing the required transmission distance between IoT-sensors and G. (*d*_*IG*_).

**Fig 6:**
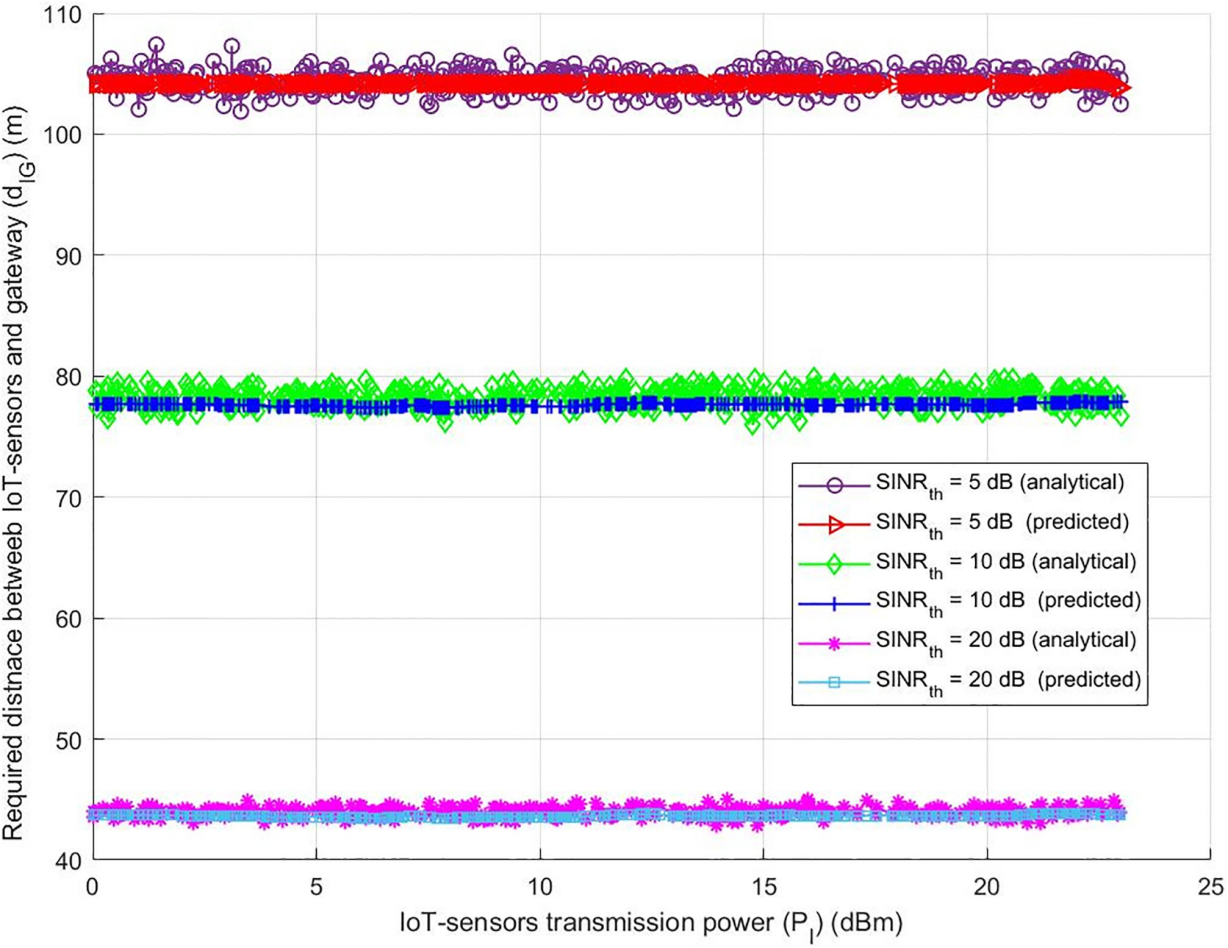
IoT-sensors transmission power (*P*_*I*_) (dBm) required distance between IoT-sensors and gateway (*d*_*IG*_) (m).

The best transmission distance between IoT-sensors and the gateway (G) is found by evaluating the system under various thresholds for the Signal-to-Interference-plus-noise-ratio (*SINR*_*th*_) (5 dB, 10 dB, and 20 dB), taking into account the various transmission power levels of IoT-sensors. It was believed that the transmission power of all interference is always equal to the transmission power of IoT sensors (*P*_*I*_). Upon closer inspection, Fig 6 displays a noteworthy trend: an increase in the transmission power of IoT-sensors. This suggests that all interfering devices in the three *SINR*_*th*_ circumstances are simultaneously seeing a rise in transmission power. Surprisingly, for both analytical and deep learning models, IoT-sensors and gateways (G) need a constant, optimal transmission distance in order to maintain system performance. This statement highlights the system’s ability to maintain performance requirements over time and how adaptable it is to variations in transmission power. It is also important to highlight that an increase in the optimal transmission distance required between IoT-sensors and gateway (G) correlates with a drop in *SINR*_*th*_. This phenomena is necessary for precise and efficient information transmission. In other words, the system compensates for *SINR*_*th*_ dips by extending the transmission distance, which maintains effective information exchange and improves the system’s overall dependability and efficiency in a range of conditions.

**Fig 7:**
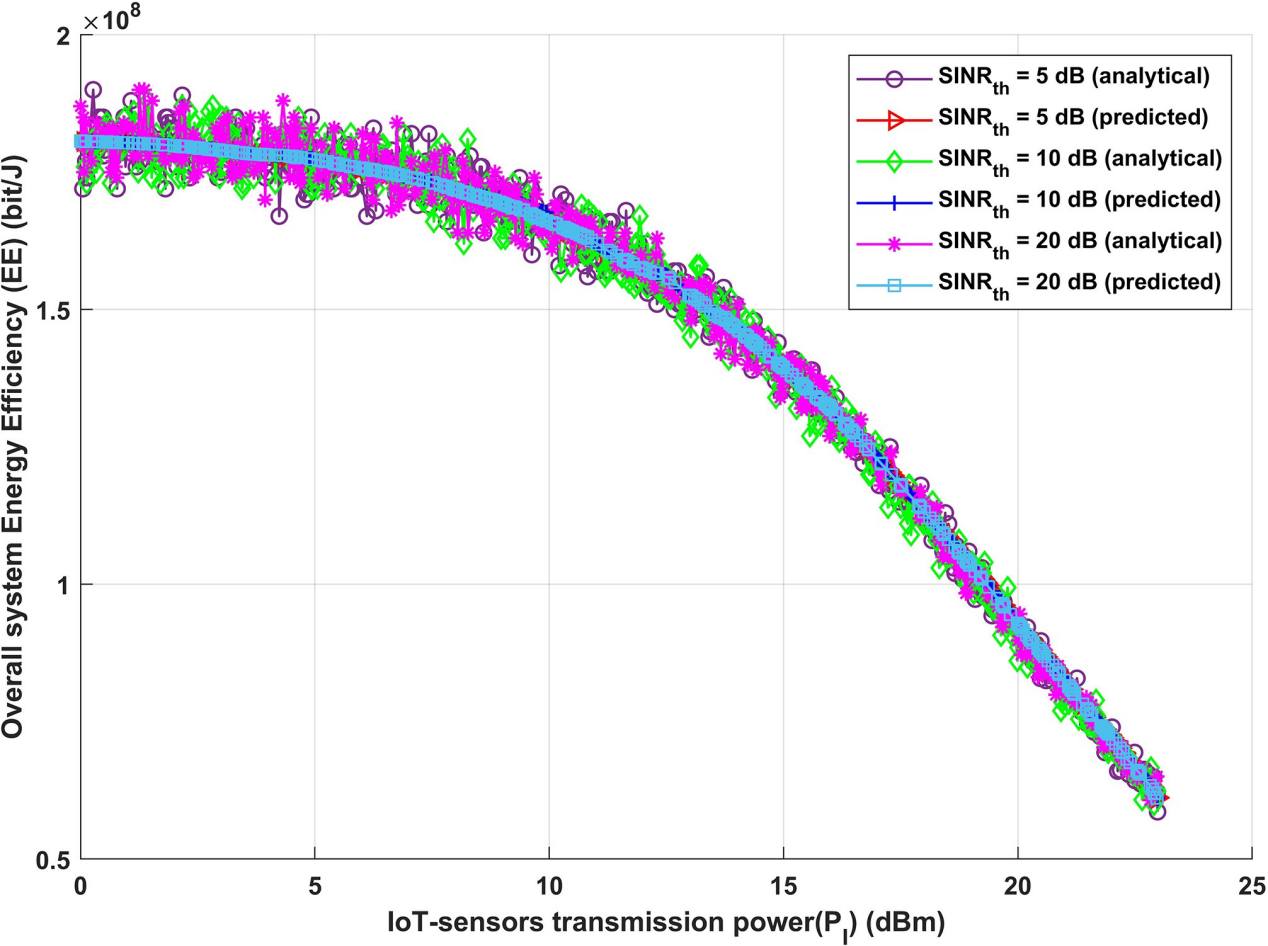
IoT-sensors transmission power (*P*_*I*_) (dBm) versus overall system energy efficiency (EE) (bit/J).

As previously noted, Fig 7 illustrates the relationship between the transmission power of IoT-sensors and the overall energy efficiency of the system for the three distinct values of *SINR*_*th*_. Here, the demonstrated decrease in energy efficiency with increasing IoT-sensors transmission power for both the analytical and deep learning models is explained by a well considered equivalency between IoT-sensors and interference transmission powers. This hypothesis states that the power of interference transmission for both analytical and deep learning models increases in direct proportion to each rise in IoT-sensors transmission power, resulting in a situation where both powers increase at the same time. This correlation introduces an important trade-off in the system dynamics. On the one hand, increasing the transmission power of IoT-sensors can help to improve signal strength and communication dependability. Nevertheless, this surge also directly causes more interference, endangering the overall efficacy of the system. It becomes evident that upholding this trade-off necessitates a careful balance, emphasizing the need for strategic decision-making in the process of selecting the optimal transmission power levels. From a practical perspective, this event highlights how important it is to consider both the benefits of increased IoT-sensor transmission power as well as the challenges posed by increased interference. System operators and designers are responsible for managing this trade-off and determining an equilibrium that maximizes energy savings without sacrificing the accuracy and dependability of information sent. As a result, the observed drop in energy efficiency is a subtle signal that warrants additional research to fully understand the complex interplay between power management and interference control within the system framework.

**Fig 8:**
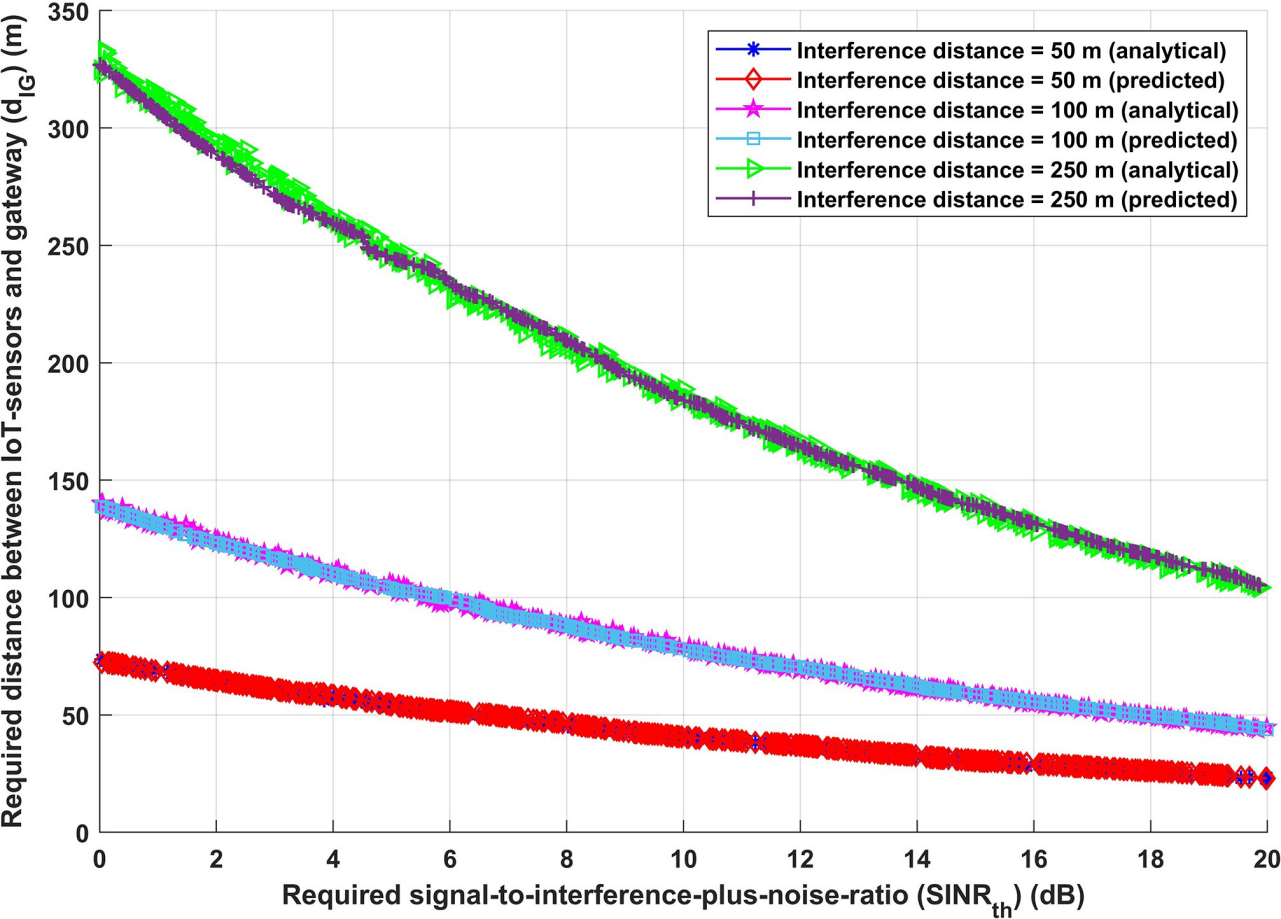
Required signal-to-interference-plus-noise-ratio (*SINR*_*th*_) versus required distance between IoT-sensors and gateway (*d*_*IG*_).

By comparing the desired distance between IoT-sensors and G (*d*_*IG*_) with the effect of all required *SINR*_*th*_, Fig 8 illustrates the efficacy and robustness of the proposed approach. Three distinct interference distances (50, 100, and 250 metres) have been established in order to assess the effectiveness of the suggested model. Additionally, it has been considered that all interfere devices and IoT sensor transmission power sent data with a maximum interfere transmission power of 23 dBm. In the worst scenario, IoT-sensor data transport may be impacted by significant amounts of interference transmission power. There is an optimal necessary transmission distance between IoT-sensors and G for both the analytical and deep learning models, for each interfere transmission distance and for interference distance given, in order to achieve the required system (*SINR*_*th*_) this is shown in Fig 8. For instance, the ideal required transmission distance between IoT-sensors and G (*d*_*IG*_) for the analytical and deep learning models, respectively, to fulfil desired system (*SINR*_*th*_), is 35.735 m and 36.562862 m when *SINR*_*th*_ is 12.2 dB and interference distance is 50 m. On the other hand, the optimum necessary transmission distance for the analytical and deep learning models, respectively, is 68.8212 m and 69.39796 m for IoT-sensors and G (*d*_*IG*_), where the interference distance is 100 m for the same specified *SINR*_*th*_. It is feasible to conclude from a comparison of the two cases that a transmission distance between IoT-sensors and G (*d*_*IG*_) is needed to accomplish the desired *SINR*_*th*_. This guarantees that the information will be received with sufficient accuracy and reliability and that it will be delivered via an effective communication route.

**Fig 9:**
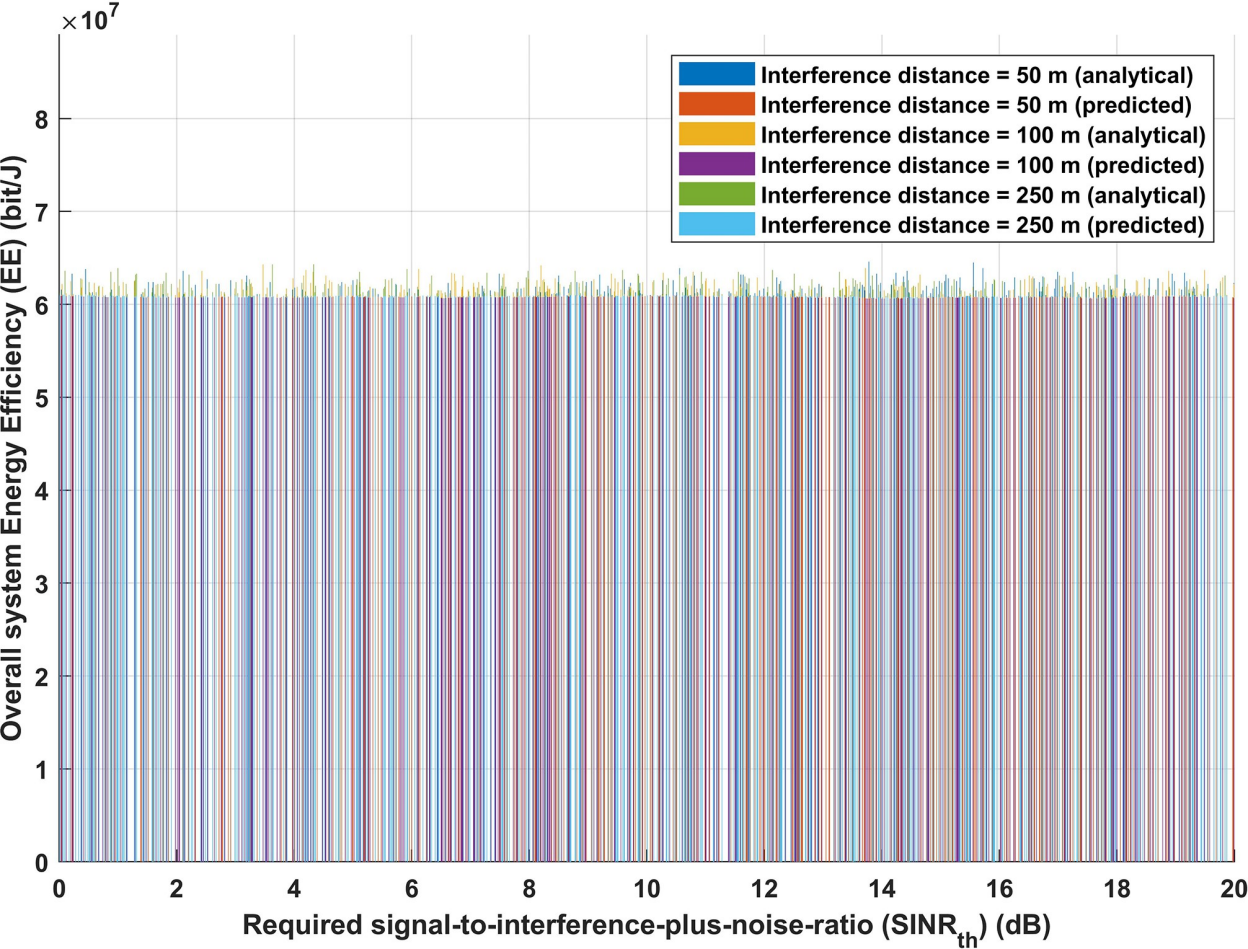
Required signal-to-interference-plus-noise-ratio (*SINR*_*th*_) versus overall system energy efficiency (EE) (bit/J).

**Fig 10:**
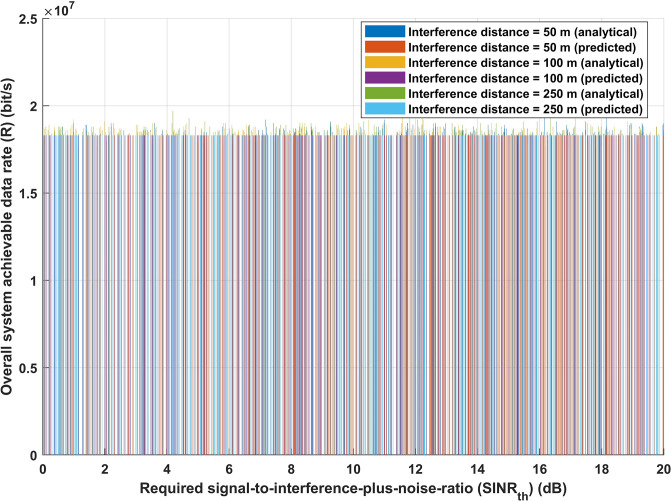
Required signal-to-interference-plus-noise-ratio (*SINR*_*th*_) versus overall system achievable data rate (R) (bit/s).

Figs 9 and 10 represent the correlation between the required signal-to-interference-plus-noise-ratio (*SINR*_*th*_) and the overall energy efficiency of the system, as well as the correlation between the required signal-to-interference-plus-noise-ratio (*SINR*_*th*_) and the overall achievable data rate. As mentioned before, three distinct interference distance values have been considered. Fig 9, using either the analytical or deep learning model, shows that there is no discernible change in the energy efficiency performance of the system when the interference distance is increased with varying values of *SINR*_*th*_. Moreover, the same performance is obtained for both analytical and deep learning models, as Fig 10 illustrates, suggesting that increasing interference distance with different values of *SINR*_*th*_ has no effect on the achievable data rate (*R*) of the system. The findings displayed in Figs 9 and 10 corroborate the idea presented in Fig 8 that altering the transmission distance in light of interference distance knowledge is one of the most crucial strategies for achieving the desired system performance. It is demonstrated that variations in the required signal-to-interference-plus-noise-ratio (*SINR*_*th*_) have minimal impact on the overall energy efficiency of the system. This resilience is proof of the proactive adjusting mechanism of IoT sensors. By dynamically selecting transmission distances, these devices confirm that the system meets the predefined performance parameters, hence validating the effectiveness of the previously established adaptive transmission method.

**Fig 11:**
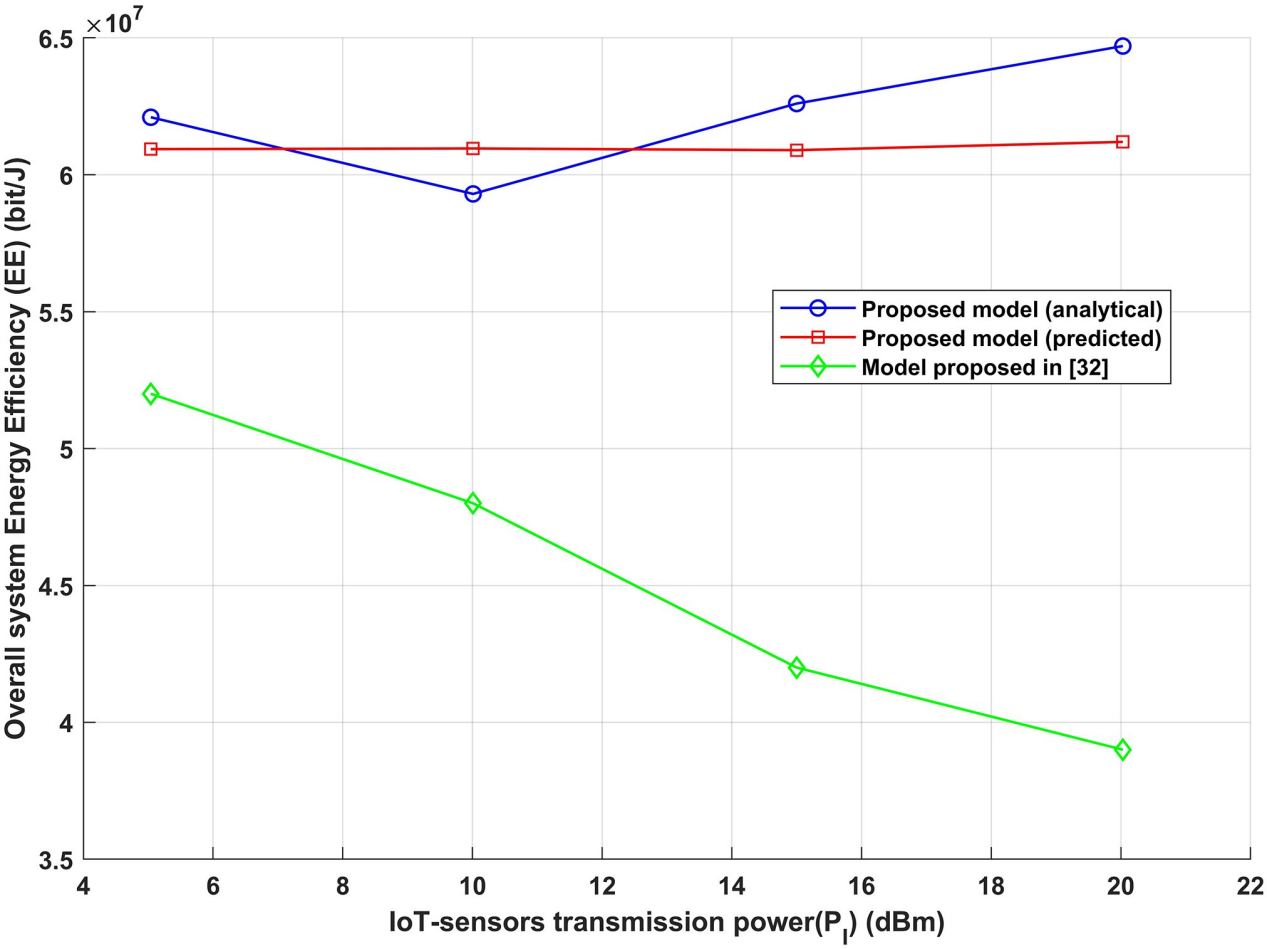
IoT-sensors transmission power (*P*_*I*_) vs overall energy efficiency (EE).

To prove the effectiveness of the proposed model, a comparison with an existing method has been made [31]. A comparison between the proposed model and the model reported in [31], which focuses on the relationship between transmission power and overall energy efficiency as shown in Fig 11, demonstrates the higher performance of this suggested strategy. The recommended approach outperforms the alternative in terms of overall energy efficiency, and this outcome can be attributed to several significant factors. First, the recommended method most likely makes use of sophisticated algorithms or methodologies to figure out the best transmission distance between IoT-sensors and G in order to maximize achievable data throughput and energy efficiency. The result of this optimization is improved energy efficiency, which allows for more efficient use of transmission power. Moreover, this adaptability enables the proposed approach to attain optimal equilibrium between signal quality and transmission range, contributing to heightened energy economy. Furthermore, energy efficiency may automatically be increased by the basic architecture or design principles of the proposed technique. This could involve new techniques for transmission protocols, interference management, or modulation methods that when combined produce a more energy-efficient system than the alternative paradigm. In summary, the proposed technique’s enhanced energy efficiency under transmission power and *SINR*_*th*_ variations which can be ascribed to its sophisticated optimization methods, versatility, and effective architecture.

## Conclusion

This paper presents a novel communication method that combines analytical and deep learning models to enable effective communication between IoT-sensors and gateways in smart farms. The issue of maximizing energy efficiency and feasible data rate has been taken into consideration in order to guarantee dependable and accurate data transmission between IoT-sensors and gateway, as well as to lessen the impact of concurrent transmission from other devices sharing the same spectrum. The quality of the data received may be impacted by such interference, which would impact the data being sent to any destination. Farmers’ decisions are impacted when the destination cannot obtain accurate data because of delayed data transmission. The first step in solving the challenge of maximizing energy efficiency and an achievable data rate is to use the Lagrange optimization technique to determine the ideal required distance between IoT-sensors and gateways. Next, this distance is simulated using MATLAB. Consequently, a recommendation was made for the recommended model for the ensuing 1D-CNN-based deep learning model. Reducing computational complexity is the aim of 1D-CNN, which makes it perfect for real-time applications and permits processing energy efficiency and overall attainable data rate. Consequently, in order to get a virtually optimal outcome, the deep learning model utilized for IoT-sensors is able to determine the ideal needed transmission distance. Therefore, the deep learning model applied to IoT-sensors can estimate the optimal necessary transmission distance to get an almost flawless result. Consequently, both approaches have been used to evaluate the optimal transmission distance between IoT-sensors and the gateway. The analytical results of anticipating the ideal required transmission distance between IoT-sensors and gateway are thus presented in order to meet the fundamental system performance requirements. Results on the achievable data rate and system energy efficiency indicate that the proposed model may perform effectively under a range of environmental circumstances. Furthermore, it has been shown through the use of analytical and deep learning techniques that a number of factors, such as the required signal-to-interference-plus-noise (*SINR*_*th*_), interfere devices transmission distance, and IoT-sensors transmission power (*P*_*I*_), can affect the required transmission distance. The findings demonstrate that, at maximum IoT-sensor and interference device power, the required signal-to-interference-plus-noise *SINR*_*th*_ climbs, as does the necessary transmission distance between IoT-sensors and gateways. This is because interference needs to be reduced or mitigated in order to get the high needed signal-to-interference-plus-noise ratio. This can be achieved by reducing the transmission data, which in turn reduces the necessary transmission distance. In the end, the findings demonstrate that the suggested model may maintain a respectable degree of efficacy and dependability while achieving the necessary IoT performance for smart farm communication.
